# Biocontrol of Rice Seedling Rot Disease Caused by *Curvularia lunata* and *Helminthosporium oryzae* by Epiphytic Yeasts from Plant Leaves

**DOI:** 10.3390/microorganisms8050647

**Published:** 2020-04-29

**Authors:** Savitree Limtong, Parichat Into, Panchapond Attarat

**Affiliations:** 1 Department of Microbiology, Faculty of Science, Kasetsart University, Bangkok 10900, Thailand; parichat_into@hotmail.com (P.I.); panchaporn.bell@gmail.com (P.A.); 2 Academy of Science, The Royal Society of Thailand, Bangkok 10300, Thailand

**Keywords:** epiphytic yeasts, rice seedling rot, biological control, *Curvularia lunata*, *Helminthosporium oryzae*, *Wickerhamomyces anomalus*, *Torulaspora indica*

## Abstract

Seedling rot disease in rice leads to significant loss in the production of seedlings. This research was conducted to explore yeasts that could be used as biological control agents against rice seedling rot disease caused by *Curvularia lunata* and *Helminthosporium oryzae.* In total, 167 epiphytic yeast strains were evaluated, revealing that 13 of these yeast strains demonstrated antagonistic activities against fungal pathogens and either *C. lunata* DOAC 2313 or *H. oryzae* DOAC 2293. The volatile organic compounds (VOCs) and biofilm produced were possible antagonistic mechanisms in vitro for all the antagonistic yeast strains. Using nursery trays in a greenhouse, this study evaluated the control of rice seedling rot disease caused by these two fungal pathogens using antagonistic yeasts, identified in the present study and from our previous study. *Torulaspora indica* DMKU-RP31 and *Wickerhamomyces anomalus* YE-42 were found to completely control rice seedling rot disease caused by both of these fungal pathogens. Furthermore, *W. anomalus* DMKU-RP04 revealed 100% disease control when the disease was caused by *H. oryzae*. This is the first report on using antagonistic yeasts to control rice seedling rot disease caused by *C. lunata* and *H. oryzae*. These three antagonistic yeasts also showed promising potential for development as biocontrol agents against rice seedling rot disease caused by fungi.

## 1. Introduction

Rice (*Oryza sativar*) is the most widely produced and consumed staple food in Asian countries [1]. In 2018, the Rice Department in Thailand’s Ministry of Agriculture and Cooperatives reported that Thailand had approximately 59.2 million hectares used for rice cultivation with rice production of 24.2 million tons [2].

In rice cultivation, seeds are germinated in nursery trays and seedlings are transplanted to fields. During rice seed germination and the growth of seedlings, various fungal pathogens may cause diseases in seeds and seedlings [3]. One of the significant diseases in rice seeds and seedlings is seedling rot, caused by various seed-borne and soil-borne fungal pathogens, such as *Curvularia lunata*, *Fusarium oxysporum*, *Helminthosporium oryzae*, *Pyricularia oryzae*, *Pythium* spp., and *Rhizoctonia solani* [2,4,5,6,7]. In Thailand, two of the significant causal agents of rice seedling rot in nursery trays are *C. lunata* and *H. oryzae*, as reported by the Rice Department, Ministry of Agriculture and Cooperatives of Thailand [2]. Rice seedling rot disease affects seeds and seedlings of rice plants, with disease symptoms often appearing within a few days of sowing the seeds. Infected seeds become soft and pulpy, and may be surrounded by white mold growth, while some cannot germinate. From the infected seeds that manage to germinate, seedlings usually turn yellow or pale green in color, are weak, grow slowly, and then die. Seedlings may also turn dark brown at the roots and base of the plant. Rice seedling rot disease leads to significant loss of seedling production [6].

The management of rice seedling rot disease caused by fungi is mainly based on the use of chemical fungicide solution in which the seeds are soaked [4,8]. However, the use of synthetic chemical fungicides is not only harmful to humans and the environment, but is also costly [8,9]. Biological control, developed by firstly exploring antagonistic microorganisms as biological control agents, is an effective and sustainable strategy for controlling plant and post-harvest diseases [10,11,12,13,14,15,16]. Microorganisms associated with plants, on both epiphytes (plant surfaces) and endophytes (inside plant tissues), are good sources of potential biocontrol agents [7,11,12,13,14,15]. Microbial antagonists may use both direct and indirect antagonistic mechanisms to control pathogens [13,14,15,16,17,18,19]. Antagonistic mechanisms include emission of antifungal volatile organic compounds (VOCs); secretion of antibiotics; production of fungal cell wall lytic enzymes; competition for nutrients and space; parasitism; and induction of localized and systemic resistance in host plants [13,14,15,16,17]. Antagonistic yeasts, using a variety of biocontrol mechanisms, have been reported to carry out biological control activities against various fungal pathogens that cause plant and post-harvest diseases [13,15,18,19]. However, only one study in literature was reported to have used antagonistic yeasts for biological control disease in rice seeds: in that study, the antagonistic yeasts, *Metschnikowia pulcherrima* and *Pichia guilliermondii*, were used to control *Fusarium fujikuroi* which causes bakanae disease in rice seeds [20].

The objective of the present research was to explore antagonistic yeasts that could be used as biological control agents against rice seedling rot disease caused by two fungal pathogens, *C. lunata* and *H. oryzae.* In this research, we evaluated the antagonistic activities of yeasts (that had been isolated from the surfaces of plant leaves) against *C. lunata* DOAC 2313 and *H. oryzae* DOAC 2293. The antagonistic yeasts obtained were tested for their antagonistic mechanisms in vivo. Lastly, using nursery trays in a greenhouse, the antagonistic yeasts, obtained from this study and our previous study, were evaluated for their efficacy in controlling rice seedling rot disease caused by these two fungal pathogens.

## 2. Materials and Methods

### 2.1. Epiphytic Yeasts

In total, 167 epiphytic yeast strains were isolated from the surfaces of rice, corn, and sugarcane leaves in Thailand and maintained in the Yeast Research Laboratory, Department of Microbiology, Faculty of Science, Kasetsart University, Thailand (Table S1). These yeast strains were grown on yeast extract malt extract (YM) (comprising 3 g/L yeast extract, 3 g/L malt extract, 5 g/L peptone, and 10 g/L glucose) agar at 25 °C.

### 2.2. Fungal Pathogens

The fungal pathogens, *C. lunata* DOAC 2313 and *Helminthosporium oryzae* DOAC 2293, that cause rice seedling rot disease were obtained from the Department of Agriculture, Ministry of Agriculture and Cooperatives, Bangkok, Thailand.

### 2.3. Evaluation In Vitro of Antagonistic Activities of Epiphytic Yeasts against Fungal Pathogens Causing Rice Seedling Rot Disease

Using dual cultivation [21] with slight modification, the 167 epiphytic yeast strains were evaluated for their antagonistic activities against *C. lunata* DOAC 2313 and *H. oryzae* DOAC 2293. For each strain, an active yeast culture obtained by cultivation on YM agar at 25 °C for two days was inoculated on potato dextrose agar (PDA) [Difco™–BBL, Sparks, MD, USA] in a Petri dish by linear streaking 3 cm from the dish edge and incubated at 25 °C for two days. A fungal mycelial plug (5 mm in diameter), obtained from an active fungal pathogen colony seven days at 25 °C on PDA agar) and cut by a cork borer, was placed on the opposite edge of the Petri dish to that previously inoculated with yeast. A PDA dish inoculated only with the fungal pathogen was used as a control. The inoculated dishes were incubated at 25 °C for seven days. Three replicates were performed for each treatment, and the experiment was repeated three times. The percentage of fungal pathogen growth inhibition was calculated as follows:

Growth inhibition (%) = (radius of fungal pathogen colony cultured alone—radius of fungal pathogen colony culture with yeast)/radius of fungal pathogen colony cultured alone × 100.

### 2.4. Evaluation of Antagonistic Mechanisms of Antagonistic Yeasts

#### 2.4.1. Production of Antifungal Volatile Organic Compounds (VOCs)

The volatile organic compound (VOC) production of the antagonistic yeasts was determined by a double dish assay [22] with slight modification, as described by Into et al. [13]. In brief, an aliquot (100 µL) of yeast cell suspension (10^8^ colony forming units [CFU]/mL) was spread on a PDA dish and incubated at 25°C for two days, while another PDA dish, inoculated with a fungal mycelial plug, was placed upside down instead of the dish cover. The control was a PDA dish inoculated only with fungal pathogens. Three replicates were performed for each treatment and control. The percentage of fungal pathogen growth inhibition was calculated as follows:

Growth inhibition (%) (diameter of fungal pathogen colony cultured alone—diameter of fungal pathogen colony culture with yeast)/diameter of fungal pathogen colony cultured alone × 100.

#### 2.4.2. Competition for Nutrients

Using the dual cultivation method described by Zhang et al. [23], the competition for nutrients was estimated by the amount of fungal pathogen growth inhibition by each antagonistic yeast in a PDA medium containing different nutrient concentrations. The evaluation was performed in the same way as in Section 2.3, but PDA media with four different nutrient concentrations were used. These consisted of: standard nutrient concentration; half of standard nutrient concentration; one-quarter of standard nutrient concentration; and one-tenth of standard nutrient concentration prepared from 39 g/L, 19.5 g/L, 9.7 g/L, and 3.9 g/L Difco™–BBL PDA powder, respectively. Three replicates for each treatment and control were carried out.

#### 2.4.3. Production of β-Glucanase and Chitinase

Production of β-glucanase and chitinase by the antagonistic yeasts was carried out by their cultivation in potato dextrose broth (PDB) [Difco™–BBL, Sparks, MD, USA] at 150 rpm and 25 °C for five days, as described by Into et al. [13]. The culture broth was collected and centrifuged at 10,000× *g* for 5 min. The supernatant was analyzed for β-glucanase and chitinase activities.

The activities of β-glucanase and chitinase were determined by the colorimetric quantification of reducing sugar and N-acetyl glucosamine (NAG) released from laminarin and colloidal chitin, respectively, as described by Into et al. [13]. The concentrations of reducing sugar and N-acetyl glucosamine were determined by Miller’s [24] method, with β-glucanase and chitinase activities expressed as units (U) per mL. One unit (U) of β-glucanase was defined as 1 µg of reducing sugar released from laminarin per minute under the assay conditions, whereas one unit (U) of chitinase was defined as 1 µg of NAG released from colloidal chitin per minute also under the assay conditions.

#### 2.4.4. Biofilm Formation

The biofilm formation of the antagonistic yeast strains was investigated using Růžička et al.’s [25] method with slight modification, as described by Into et al. [13]. In brief, an aliquot (20 µL) of yeast cell suspension (cells grown on PDA at 25 °C for two days suspended in sterile water and adjusted to an optical density (OD) measured at 600 nm of 0.5) was inoculated into each well of a 96-well microtiter plate containing 180 µL of PDB. The microtiter plate was incubated at 25 °C for 48 h (h). A well containing only PDB was used as a negative control. Three replicates were performed for each treatment. After 48 h, the wells were emptied, rinsed with water, and air-dried at room temperature. The adherent biofilm layer was stained with an aqueous solution of 1% (*w*/*v*) crystal violet for 20 min, rinsed with water, and air-dried. The stained biofilm layer was eluted from each well with 200 µL of ethanol, and the OD of each well was measured at 620 nm. Biofilm formation in a well was regarded as positive when the mean OD of the treatment was higher than the mean OD of the negative control (ODc). The following classification was applied in the determination of biofilm formation: weak biofilm producer (ODc < ODt ≤ 2 ODc); moderate biofilm producer (2 ODc < ODt ≤ 4 ODc); and strong biofilm producer (4 ODc < ODt) [26].

#### 2.4.5. Siderophore Production

Siderophore production by the antagonistic yeasts was investigated by their cultivation on chrome azurol S (CAS) [Sigma-Aldrich Chemie GmbH, Schnelldorf, Germany] blue agar in a Petri dish [27]. An aliquot of yeast cell suspension was prepared by suspending a loopful of culture grown on YM agar at 25 °C for two days in 3 mL of 0.85% sterile normal saline, adjusted to an OD_600_ of 0.10. An aliquot (10 µL) of yeast cell suspension was dropped onto the surface of CAS blue agar and incubated in the dark at 25 °C for 10 days. Three replicates were performed for each treatment. Siderophore production was confirmed by a change in the color of the medium from blue to purple or yellow around the yeast colony.

#### 2.4.6. Phosphate and Zinc Oxide Solubilization

Phosphate and zinc oxide solubilization by the antagonistic yeasts was determined by their cultivation on Pikovskaya’s agar [28] and zinc oxide agar [29], respectively. An aliquot (5 µL) of yeast cell suspension (OD_600_ of 0.10), prepared in the same way as in Section 2.4.5, was dropped onto the surface of Pikovskaya’s agar or zinc oxide agar in a Petri dish and incubated at 25 °C for five days. Three replicates were performed for each treatment. The solubilization efficiency (SE) of the phosphate and zinc oxide was designed as a ratio of the halo zone diameter and the colony diameter, with both these diameters measured.

### 2.5. Evaluation of the Efficacy of Antagonistic Yeasts in Controlling Rice Seedling Rot Disease in a Greenhouse

In this study, yeast strains, which showed antagonistic activities against *C. lunata* DOAC 2313 and/or *H. oryzae* DOAC 2293, were evaluated for their efficacy in the control of rice seedling rot disease caused by *C. lunata* or *H. oryzae*. In addition, from the previous study, the other nine yeast strains were subjected for the same evaluation. They consisted of four strains (*Torulaspora indica* DMKU-RP31, *T. indica* DMKU-RP35, *Wickerhamomyces anomalus* DMKU-RP04 and *W. anomalus* DMKU-RP25), which revealed antagonistic activities against both fungal pathogens, five strains (*Kodamaea ohmeri* DMKU-RP06, *K. ohmeri* DMKU-RP57, *K. ohmeri* DMKU-RP233 and *Meyerozyma* *caribbica* DMKU-RP07) that inhibited only *C. lunata* DOAC 2313, and one strain (*M. caribica* DMKU-RP55), which inhibited *H. oryzae* DOAC 2293 [13].

In the present study, the Thai Jasmine rice cultivar, Khao Dawk Mali 105, was used. Rice seeds (15 g) were surface sterilized by 100 mL of 10% Clorox solution for one minute, rinsed with sterile distilled water, and air dried at room temperature.

Each antagonistic yeast was cultivated in yeast extract peptone dextrose (YPD) broth (comprised of 20 g/L glucose, 20 g/L peptone, and 10 g/L yeast extract) on a rotary shaker at 150 rpm, at 25 °C for 48 h (h). Yeast cell suspension was prepared by suspending cells collected from the culture broth by centrifugation at 5000× *g* for 10 min in the sterile Ringer solution. Cells were quantified with a hemacytometer to reach a concentration of 10^8^ cells/mL.

Fungal pathogens were grown on PDA at 25 °C for seven days, with the spore suspension (10^5^ spores/mL) prepared in sterile 0.05% Tween 20. Soil was put in a plastic bag and sterilized in an autoclave at 121 °C for 30 min. The sterilization was performed twice.

Infected rice seed was prepared, as described by Khalili et al. [12]. Sterilized rice seeds were soaked in the spore suspension (10^5^ spores/mL) of each fungal pathogen and then shaken on a rotary shaker (TAITAC BR-300LF, Saitama, Japan), at 100 rpm, at room temperature (28–32 °C) for 24 h and then air-dried. The infected seeds were soaked in the yeast cell suspension (10^8^ cells/mL) for two hours; 100 treated seeds were then transplanted into a plastic tray (20 cm wide × 35 cm long × 10 cm high) containing sterile soil (4 kg). The seeds were sown in four rows: in each row, 16–17 seeds were placed, with each seed 2–3 cm apart from the other seeds. The tray was incubated in greenhouse conditions (11 h light/13 h dark, and at 30–34 °C day/20–24 °C night) for 45 days. Three replicates were performed for each treatment (3 × 100 seeds). The number of germinated seeds, and seedlings’ stem height, root length and dry weight were recorded.

The percentages of seed germination, seedling vigor index (SVI), disease incidence and disease control were calculated as follows:

Seed germination (%) = (number of germinated seeds/total number of seeds planted) × 100
Seedling vigor index (SVI) (%) = (stem height + root length) × seed germination (%)
Disease incidence (%) = [(SVI of negative control − SVI of positive control or treatment)/SVI of negative control] × 100
Disease control (%) = [(disease incidence of positive control − disease incidence of each treatment)/disease incidence in positive control] × 100

### 2.6. Statistical Analysis

Data were analyzed with statistical analysis software IBM^®^ SPSS Statistics version 22 (Armonk, NY, USA). All data were first subjected to analysis of variance (ANOVA). Means were compared using Duncan’s multiple range test and a significance level of *p* ≤ 0.05 was considered as being significantly different.

## 3. Results

### 3.1. Evaluation in Vitro of Antagonistic Activities of Epiphytic Yeasts against Fungal Pathogens Causing Rice Seedling Rot Disease

**Figure 1:**
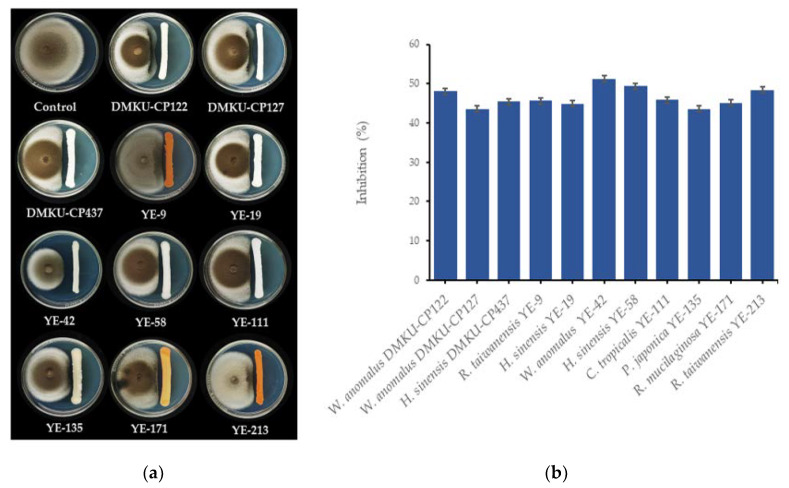
(**a**) Growth on potato dextrose agar (PDA) at 25 °C for seven days of *Curvularia lunata* DOAC 2313 alone, and dual cultured with *Wickerhamomyces anomalus* DMKU-CP122, *W. anomalus* DMKU-CP127, *Hannaella sinensis* DMKU-CP437, *Rhodotorula taiwanensis* YE-9, *H. sinensis* YE-19, *W. anomalus* YE-42, *H. sinensis* YE-58, *Candida tropicalis* YE-111, *Papiliotrema japonica* YE-135, *Rhodotorula mucilaginosa* YE-171, or *R. taiwanensis* YE-213; and (**b**) inhibition of growth of *C. lunata* DOAC 2313 by 11 antagonistic yeast strains determined by dual cultivation on PDA at 25 °C for seven days.

**Figure 2:**
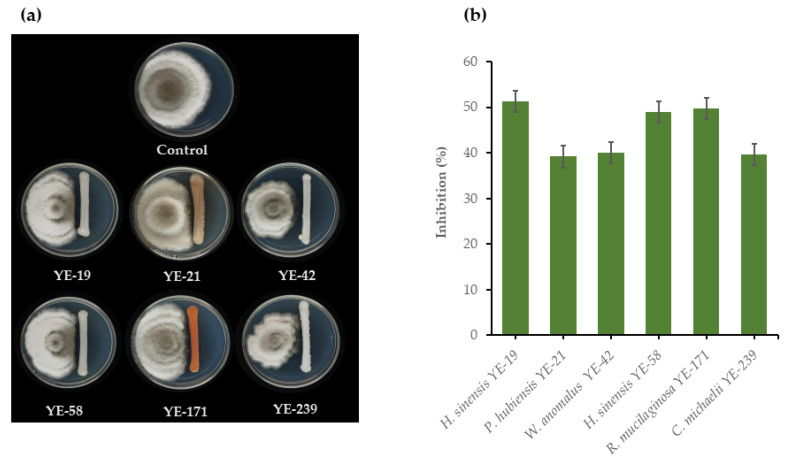
(**a**) Growth on PDA at 25 °C for seven days of *Helminthosporium oryzae* DOAC 2293 alone, and dual cultured with *Hannaella sinensis* YE-19, *Pseudozyma hubiensis* YE-21, *Wickerhamomyces anomalus* YE-42, *H. sinensis* YE-58, *Rhodotorula mucilaginosa* YE-171 and *Candida michaelii* YE-239; and (**b**) Inhibition of *H. oryzae* DOAC 2293 growth by six antagonistic yeast strains determined by dual cultivation on PDA at 25 °C for seven days.

Evaluation of the antagonistic activities of 167 epiphytic yeasts against two rice seedling rot fungal pathogens, *C. lunata* DOAC 2313 and *H. oryzae* DOAC 2293, revealed that only 13 yeast strains from eight species possessed these antagonistic activities. These species were *Candida michaelii* (one strain), *Candida tropicalis* (one strain), *Hannaella sinensis* (three strains), *Papiliotrema japonica* (one strain), *Pseudozyma hubiensis* (one strain), *Rhodotorula mucilaginosa* (one strain), *Rhodotorula taiwanensis* (two strains), and *W. anomalus* (three strains). Of the antagonistic yeast strains, 11 inhibited the growth of *C. lunata* DOAC 2313 by 43.5%–51.2%, while *W. anomalus* YE-42 was the antagonist with the strongest inhibitory effect (Figure 1). Six antagonistic yeasts inhibited the growth of *H. oryzae* DOAC 2293 by 39.2%–51.2%, with *H. sinensis* YE-19 the antagonist with the strongest inhibitory effect (Figure 2). Moreover, four yeast strains, namely, *H. sinensis* YE-19, *H. sinensis* YE-58, *R. mucilaginosa* YE-171, and *W. anomalus* YE-42, inhibited both fungal pathogens that caused rice seedling rot disease (Figure 1 and Figure 2).

### 3.2. Evaluation of Antagonistic Mechanisms of Antagonistic Yeasts

#### 3.2.1. Production of Antifungal Volatile Organic Compounds (VOCs)

The present study evaluated the antagonistic activities against *C. lunata* DOAC 2313 and *H. oryzae* DOAC 2293 by VOCs production form 11 and six antagonistic yeast strains, respectively. The results revealed that all antagonistic yeast strains produced VOCs inhibited growth of their related fungal pathogens but with lower inhibition percentages than using yeast cultures (Table 1). The 10 antagonistic yeast strains tested inhibited *C. lunata* DOAC 2313 growth by 4.2–32.1% and *W. anomalus* YE-42 produced VOCs that showed the strongest inhibitory effect on this fungal pathogen. On the other hand, the six antagonistic yeast strains tested with *H. oryzae* DOAC 2293 produced VOCs inhibited this fungal pathogen in the range of 0.7% to 25.1% and *W. anomalus* YE-42 revealed the strongest antifungal VOCs activity. However, *H. sinensis* YE-19, which showed the strongest inhibitory effect when tested by dual cultivation with *H. oryzae* DOAC 2293 produced VOCs inhibited this fungal pathogen only by 1%.

#### 3.2.2. Production of β-Glucanase and Chitinase

The production of β-glucanase and chitinase by the 13 antagonistic yeast strains was determined by their cultivation in PDB at 25 °C for five days with observation of the enzymatic activities of cell-free culture broth. The results revealed that β-glucanase was produced by 11 strains: however, only a small quantity (0.2–116.5 mU/mL) was produced, with *R. mucilaginosa* YE-171 producing the highest amount (Table 2). Eight antagonistic yeast strains produced small amounts (1.0–504.5 mU/mL) of chitinase, while *P. japonica* YE-135 produced the largest amount.

#### 3.2.3. Biofilm Formation

The biofilm formation of the 13 antagonistic yeast strains was determined, with the results indicating that all 13 strains formed biofilm. Of the 13 strains, eight revealed moderate biofilm formation, while four showed weak biofilm formation. The only strain with strong biofilm formation was *C. tropicalis* YE-111 (Table 3).

#### 3.2.4. Siderophore Production

**Figure 3:**
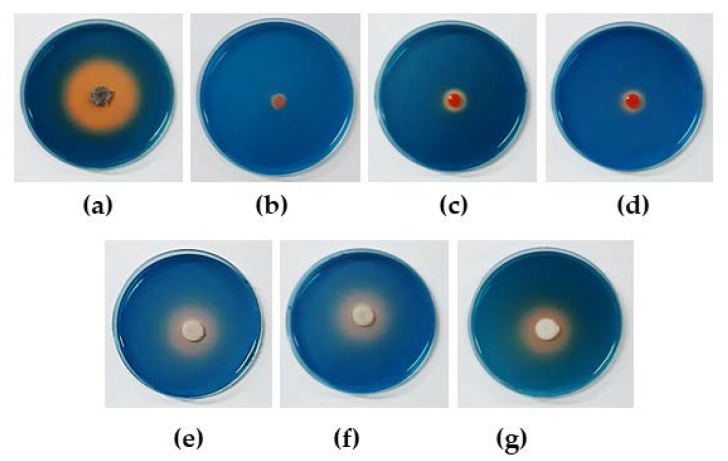
Siderophore production by antagonistic yeasts on chrome azurol S blue agar incubated for 10 days; (**a**) *Pseudozyma hubiensis* YE-21; (**b**) *Rhodotorula mucilaginosa* YE-171; (**c**) *Rhodotorula taiwanensis* YE-9; (**d**) *R. taiwanensis* YE-213; (**e**) *Wickerhamomyces anomalus* DMKU-CP122; (**f**) *W. anomalus* DMKU-CP127; and (**g**) *W. anomalus* YE-42.

Determination of the siderophore production of the antagonistic yeast strains on chrome azurol sulfonate (CAS) agar dishes revealed that seven strains, namely *P. hubiensis* YE-21, *R. mucilaginosa* YE-71, *R. taiwanensis* YE-9, *R. taiwanensis* YE-213, *W. anomalus* DMKU-CP122, *W. anomalus* DMKU-CP127, and *W. anomalus* YE-42, formed orange halo zones (9.1–46.5 mm) around the colony (Table 3, Figure 3). This result indicated that these seven antagonistic yeast strains produced siderophores.

#### 3.2.5. Phosphate and Zinc Oxide Solubilization

**Figure 4:**
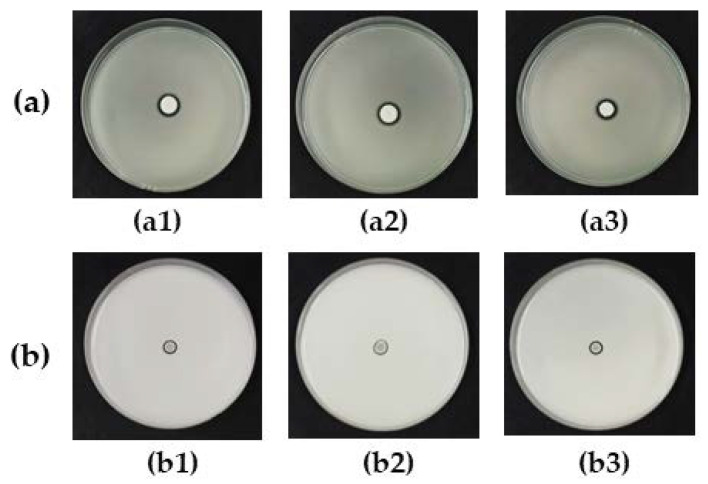
Solubilization of (**a**) phosphate on Pikovskaya’s agar and (**b**) zinc oxide on zinc oxide agar incubated for 5 days by antagonistic yeasts (a1,b1) *Wickerhamomyces anomalus* DMKU-CP122; (a2,b2) *W. anomalus* DMKU-CP127; and (a3,b3) *W. anomalus* YE-42.

The phosphate and zinc oxide solubilizing activity of all antagonistic yeasts were determined on Pikovskayaʹs agar and zinc oxide agar, respectively, showing that only three strains of *W. anomalus* grew and produced halo zones around colonies. The phosphate and zinc oxide solubilization efficiency (SE) units were in the range of 1.3–1.5 (Table 3, Figure 4).

### 3.3. Evaluation of the Control of Rice Seedling Rot Disease in a Greenhouse

The efficacy of antagonistic yeasts, obtained from the present study and the previous study, in the control of rice seedling rot disease caused by *C. lunata* DOAC 2313 and *H. oryzae* DOAC 2293 were evaluated in nursery trays in a greenhouse. The two chemical fungicides, carbendazim^®^ and mancozeb^®^, were used for comparison.

**Figure 5:**
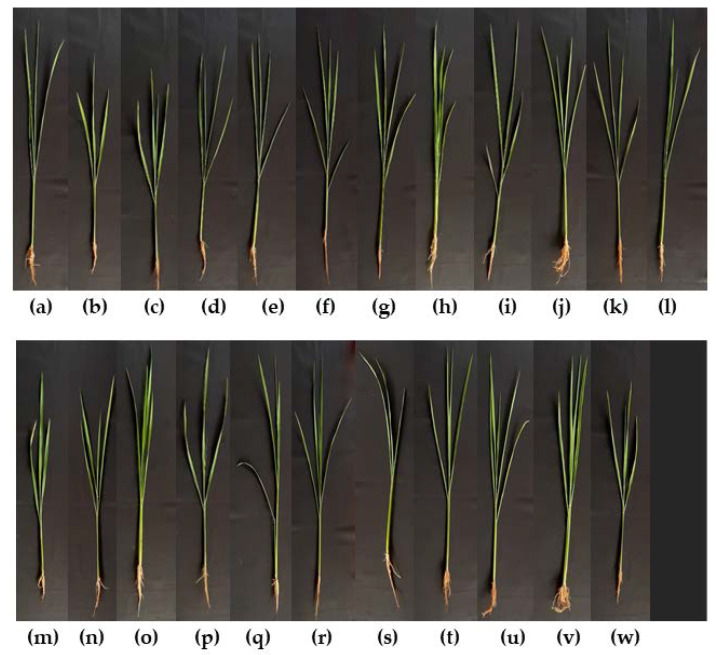
Rice seedlings (45-day-old) obtained from (**a**) seed treated with sterile water (negative control); (**b**) seed inoculated with *C. lunata* DOAC 2313 alone (positive control); and seed inoculated with *C. lunata* DOAC 2313 (**c**) with *C. tropicalis* YE-111; (**d**) with *H. sinensis* YE-19; **(e**) with *H. sinensis* YE-58; (**f**) with *H. sinensis* DMKU-CP437; (**g**) with *K. ohmeri* DMKU-RP06; (**h**) with *K. ohmeri* DMKU-RP57; (**i**) with *K. ohmeri* DMKU-RP233; (**j**) with *M. caribica* DMKU-RP07; (**k**) with *P. japonica* YE-135; (**l**) with *R. mucilaginosa* YE-171; (**m**) with *R. taiwanensis* YE-9; (**n**) with *R. taiwanensis* YE-213; (**o**) with *T. indica* DMKU-RP31; (**p**) with *T. indica* DMKU-RP35; (**q**) with *W. anomalus* DMKU-CP122; (**r**) with *W. anomalus* DMKU-CP127; (**s**) with *W. anomalus* DMKU-RP04; (**t**) with *W. anomalus* DMKU-RP25; (**u**) with *W. anomalus* YE-42; (**v**) with carbendazim^®^; or (**w**) with mancozeb^®^.

To test the control of rice seedling rot disease caused by *C. lunata* DOAC, 19 antagonistic yeast strains were tested. *T. indica* DMKU-RP31 and *W. anomalus* YE-42 were found to show higher seedling vigor index (SVI) percentages than the negative control and carbendazim^®^: these two strains resulted in no disease incidence and, consequently, 100% disease control (Table 4, Figure 5). Fourteen of the 19 antagonistic yeast strains showed no significantly different SVI percentages when compared with the negative control and carbendazim^®^: they resulted in disease incidence ranging from 2.17%–16.57% and, consequently, disease control of 45.70%–92.88%. The remaining four yeast strains revealed lower SVI percentages than the negative control and carbendazim^®^: they resulted in low disease control ranging from 33.19%–42.50%. When using the two chemical fungicides, carbendazim^®^ and mancozeb^®^, the study revealed 6.27% and 30.48% disease incidence and, consequently, 79.47% and 0.15% disease control, respectively.

**Figure 6:**
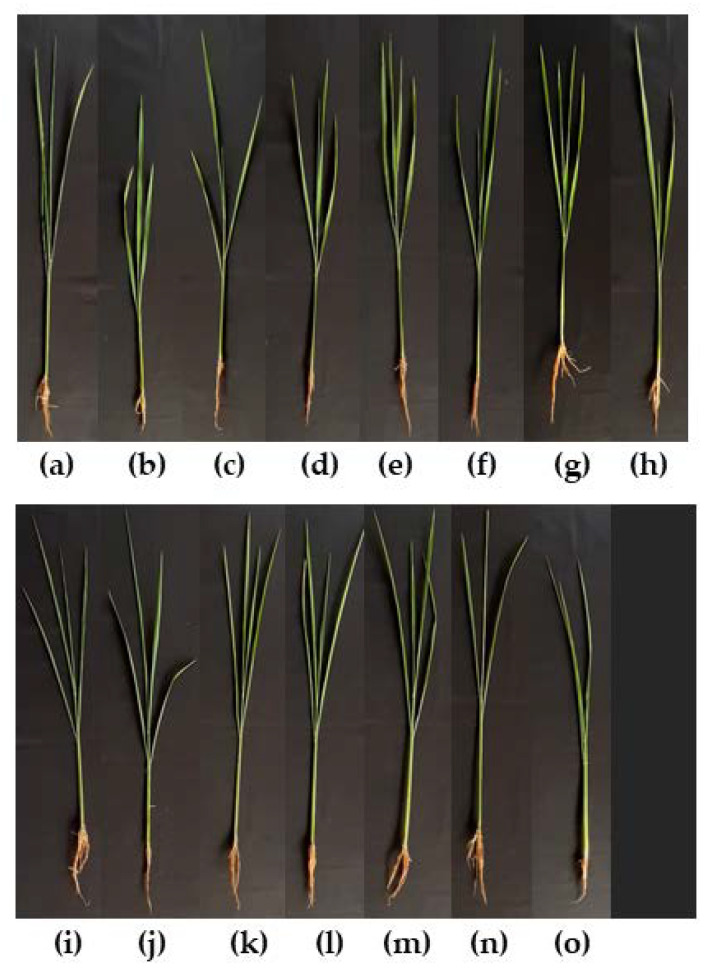
Rice seedlings (45-day-old) obtained from (**a**) seed treated with sterile water (negative control); (**b**) seed inoculated with *H. oryzae* DOAC 2293 alone (positive control); and seed inoculated with *H. oryzae* DOAC 2293 (**c**) with *C. michaelii* YE-239; (**d**) with *H. sinensis* YE-19; (**e**) with *H. sinensis* YE-58; (**f**) with *M. caribica* DMKU-RP55; (**g**) with *P. hubiensis* YE-21; (**h**) with *K. ohmeri* DMKU-RP57; (**i**) with *R. mucilaginosa* YE-171; (**j**) with *T. indica* DMKU-RP31; (**k**) with *P. japonica* YE-135; (**l**) with *W. anomalus* DMKU-RP04; (**m**) with *W. anomalus* YE-42; (**n**) with carbendazim^®^; or (**o**) with mancozeb^®^.

Evaluation of 11 antagonistic yeast strains was conducted to investigate if they had an inhibitory effect on *H. oryzae* DOAC 2293 to control rice seedling rot disease which it caused. *T. indica* DMKU-RP31, *W. anomalus* DMKU-RP04, and *W. anomalus* YE-42 showed higher SVI percentages than the negative control and carbendazim^®^; these strains resulted in no disease incidence and, consequently, 100% disease control (Table 5, Figure 6). Six antagonistic strains revealed no SVI percentages that were significantly different and, consequently, disease control ranged from 75.27%–97.45%. The remaining three strains showed lower SVI percentages than the negative control and carbendazim^®^ and, consequently, 44.95%–63.37% disease control. Mancozeb^®^ revealed the lowest disease control (of 3.05%) whereas carbendazim^®^ showed a high level of disease control at 94.04%.

## 4. Discussion

The results of this study revealed that the yeast species showing antagonistic activity against the two fungal pathogens, *C. lunata* DOAC 2313 and *H. oryzae* DOAC 2293, the causes of rice seedling rot disease, were from both yeast phyla, namely, phyla Ascomycota (*C. michaelii*, *C. tropicalis*, and *W. anomalus*) and Basidiomycota (*H. sinensis*, *P. japonica*, *P. hubeiensis*, *R. mucilaginosa*, and *R. taiwanensis*). Some antagonistic yeast species found in the present study have previously been reported to possess antagonistic activities. *W. anomalus* was reported for its ability to antagonize various fungal pathogens, the causes of plant and post-harvest diseases, such as *Alternaria alternata*, *Aspergillus carbonarius*, *C. lunata*, *Botrytis cinerea*, *Fusarium moniliforme*, *Helmintosporium oryzae*, *Monilinia fructicola*, *Penicillium expansum*, *Rhizoctonia solani*, *Cladosporium* spp., and *Colletotrichum* spp. [14,30,31]. *C. tropicalis* revealed the ability to control black rot in pineapple caused by *Chalara paradoxa* [32]. *Cryptococcus albidus* was found to be the effective antagonistic yeast against *P. expansum* and apple blue mold [30]. *H. sinensis* and *R. taiwanensis* were reported for their antagonistic activities against *Aspergilus flavus* [33,34]. *R. mucilaginosa* demonstrated the ability to antagonize *Botrytis cinerea*, the cause of gray mold spoilage in strawberries [35] and *Penicillium expansum*, the cause of post-harvest apple disease [36]. *P. hubeiensis* could control *Lasiodiplodia theobromae* [15].

In the present study, the antagonistic mechanisms of 13 yeast strains from eight species were evaluated in vitro, with the results revealing that all strains possessed the ability to produce antifungal VOCs and biofilm to antagonize *C. lunata* and *H. oryzae*. Some strains produced both of the cell wall lytic enzymes, β-glucanase and chitinase, some strains produced only β-glucanase, whereas some did not produce either enzyme. The production of cell wall lytic enzymes appeared to be a strain-specific characteristic, not a species-specific characteristic. The antagonistic yeast strains of four species, namely, *P. hubiensis*, *R. mucilaginosa*, *R. taiwanensis*, and *W. anomalus* produced siderophores, with *W. anomalus* also capable of phosphate and zinc oxide solubilization. Therefore, the antagonistic mechanisms of all the yeast strains were found to emit antagonistic antifungal VOCs and to form biofilm. Some yeast strains could possibly have additional mechanisms.

As shown in the present study, the production of antifungal VOCs, which are low molecular weight chemical compounds with high vapor pressure, played a role in the antagonistic activity of *C. michaelii*, *C. tropicalis*, *H. sinensis*, *P. japonica*, *P. hubeiensis*, *R. mucilaginosa*, *R. taiwanensis*, and *W. anomalus.* This result was in agreement with other investigators’ results which showed that the emission of VOCs by antagonistic yeasts has proven to be one of the important direct antagonistic mechanisms against various fungal pathogens of antagonistic yeasts [14,33,37,38,39]. *Debaryomyces nepalensis* produced VOCs to control the pathogen *Colletotrichum gloeosporioides* in mango fruit [40]. *Metschnikowia pulcherrima* was found to produce VOCs that could suppress the growth of *C. gloesporioides* [41]. *T. indica* and *P. hubeiensis* were reported to produce VOCs that inhibited the growth of *L. theobromae*, whereas *P. aspenensis* produced VOCs that could inhibit the growth of *C. gloeosporioides* [15]. *W. anomalus*, *M. pulcherrima*, and *S. cerevisiae* produced VOCs capable of decreasing the mycelial growth of *B. cinerea*, *M. fructicola*, *A. alternata*, *A. carbonarius*, *P. digitatum*, *Cladosporium* spp., and *Colletotrichum* spp. [31]. *W. anomalus* produced VOCs which inhibited the growth of *C. lunata*, *F. moniliforme*, and *R. solani*, whereas *K. ohmeri* produced VOCs which inhibited only the growth of *R. solani* [14]. *T. indica* produced VOCs to strongly inhibit the growth of *R. solani* and *Pyricularia oryza* [13].

In this study, no antagonistic yeasts had the characteristic of competition for nutrients when dual cultured with *C. lunata* DOAC 2313 and *H. oryzae* DOAC 2293. However, some yeast species were reported to compete for nutrients with the pathogens. For example, with *P. hubeiensis* and *T. indica*, it was revealed that competition for nutrients resulted in inhibition in the growth of *L. theobromae* [40]. *M. pulcherrima* was reported to control *C. gloeosporioides* through its ability to compete for nutrients [41].

All the antagonistic yeast strains tested could form biofilm to different degrees. This means that competition for space was one of the antagonistic mechanisms of these antagonistic yeasts. Competition for space is based on biofilm formation. Some yeasts form biofilm, allowing them to adhere to surfaces, colonize, and resist stresses [42]. *W. anomalus* and *P. hubeiensis* were previously reported to form biofilm [14,15,43]. In addition, some other yeast species were reported to form biofilm that played a role in biological control activities, such as *K. ohmeri*, *Pichia fermentans*, *P. aspenensis*, and *T. indica* [15,44].

The results of the present study revealed that β-glucanase and chitinase were produced by some strains of *C. michaelii*, *C. tropicalis*, *H. sinensis*, *P. japonica*, *P. hubiensis*, *R. mucilaginosa*, *R. taiwanensis*, and *W. anomalus*. This indicated that the fungal cell wall lytic enzymes of some strains of these yeast species could be one of the mechanisms for controlling rice seedling rot fungal pathogens. Some antagonistic yeasts were reported to produce cell wall lytic enzymes which could play a role in antagonistic activity, for example, *W. anomalus*, *K. ohmeri*, *M. caribbica*, *Meyerozyma guilliermondii*, *M. pulcherrima*, and *T. indica* [13,45,46,47].

Siderophores are involved in competition for the nutrient iron (Fe^+3^) [48]. When antagonistic microorganisms have this ability, they can use the iron; therefore, the iron in the substrate is depleted, resulting in limited growth of the pathogens. The result of this study showed that seven antagonistic yeast strains in four species, *P. hubiensis*, *R. mucilaginosa*, *R. taiwanensis*, and *W. anomalus*, produced siderophores. Therefore, producing siderophores could be one of the antagonistic mechanisms of these antagonistic yeasts. *P. hubeiensis* and *W. anomalus* have previously been reported to produce siderophores [13,14,15]. In addition, other yeast species were found to produce siderophores, such as *P. aspenensis*, *Rhodotorula glutinis*, and *T. indica* [15,49].

Solubilization of phosphate and zinc oxide can convert them to a soluble form, easily assimilated by plants, that promotes plant growth [50,51]. The results of the present study indicated that phosphate and zinc oxide solubilization was among the indirect mechanisms of *W. anomalus*, with strains of this species having previously been reported to have the ability to solubilize phosphate and zinc oxide [13,14].

The evaluation of the control of rice seedling rot disease caused by *C. lunata* DOAC 2313 and *H. oryzae* DOAC 2293 revealed that all the antagonistic yeast strains tested could control this disease. Of these antagonistic yeasts, *T. indica* DMKU-RP31, obtained from Into et al.’s [13] previous study, and *W. anomalus* YE-42, derived in the present study, could completely control the disease caused by both fungal pathogens. On the other hand, *W. anomalus* DMKU-RP04, obtained from the previous study, could completely control the disease only when infected with *C. lunata* DOAC 2313. These three yeast species demonstrated better control of rice seedling rot disease than the effective chemical fungicide, carbendazim^®^. Interestingly, *T. indica* DMKU-RP31 was recently reported to control fruit disease in post-harvest ripe mangos caused by *Lasiodiplodia theobromae* [15].

Reports on the management of diseases in rice seed and seedlings to date are limited. Chemical fungicides have been used to control rice seed and seedling diseases caused by different fungal pathogens, with these including carbendazim^®^, difolatan^®^, mancozeb^®^, propiconazole^®^, and validamycin^®^ [52,53,54,55,56]. In the present study, two chemical fungicides, carbendazim^®^ and mancozeb^®^, were used for comparison with antagonistic yeasts in the control of rice seedling rot disease caused by *C. lunata* and *H. oryzae*. Mancozeb^®^ was found to have very weak controlling ability, 0.15% and 3.05% disease control, for *C. lunata* and *H. oryzae*, respectively. The reason may be that mancozeb^®^ inhibited rice seed germination [57]. The management of rice seedling rot disease by using soil amended with tricin-releasing rice hulls was reported, with this showing suppression of the soil-borne fungal pathogens viz. *F. oxysporum* and *R. solani* that caused rice seedling rot disease [Kong et al., 2010]. The reason was that ricin was reported as being detected in rice hulls and that it had fungicidal activity. Few reports were found on using antagonistic microorganisms to control rice seedling rot disease. Research has explored the use of antagonistic bacteria, *Pseudomonas fluorescens* and *Pseudomonas tolaasii*, to control rice seedling rot disease caused by *Achlya klebsiana* and *Pythium spinosum* [11]. However, for yeasts, the only report found used *Metschnikowia pulcherrima* and *Pichia guilliermondii* to control *Fusarium fujikuroi* caused by bakanae disease in rice seeds [20]. To the best of our knowledge, the report on the present study is the first one on the use of yeasts for the control of rice seedling rot disease caused by *C. lunata* and *H. oryzae*.

## Figures and Tables

**Table 1:** Growth inhibition of *Curvularia lunata* DOAC 2313 and *Helminthosporium oryzae* DOAC 2293 by antifungal volatile organic compounds (VOCs) produced by antagonistic yeasts, on PDA containing different nutrient concentrations.

Fungal Pathogen and Yeast	Growth Inhibition by VOCs (%) ^1^	Growth Inhibition on PDA with Different Nutrient Concentration ^2^			
		A ^3^	B ^4^	C ^5^	D ^6^
*Curvularia lunata* DOAC 2313 with					
*Candida tropicalis* YE-111	20.1 ± 1.4c	45.9 ± 2.4a	25.2 ± 2.0b	0c	0c
*Hannaella sinensis* DMKU-CP437	14.3 ± 1.6f	45.4 ± 1.3a	17.1 ± 1.3b	0c	0c
*Hannaella sinensis* YE-19	17.4 ± 0.8de	44.8 ± 0.6a	17.9 ± 0.7b	0c	0c
*Hannaella sinensis* YE-58	5.5 ± 2.2h	49.3 ± 0.2a	21.8 ± 1.6b	0c	0c
*Papiliotrema japonica* YE-135	4.2 ± 0.8h	43.6 ± 1.9a	20.1 ± 1.6	0c	0c
*Rhodotorula mucilaginosa* YE-171	10.4 ± 0.9g	45.1 ± 2.0a	19.4 ± 1.0b	0c	0c
*Rhodotorula taiwanensis* YE-9	32.1 ± 1.0b	45.6 ± 1.9a	17.4 ± 0.8b	0c	0c
*Rhodotorula taiwanensis* YE-213	31.8 ± 1.0b	48.4 ± 0.5a	19.0 ± 5.6	0c	0c
*Wickerhamomyces anomalus* DMKU-CP122	19.8 ± 0.9cd	48.1 ± 1.4a	21.2 ± 1.1b	6.1 ± 1.2c	0d
*Wickerhamomyces anomalus* DMKU-CP127	15.3 ± 1.4ef	43.5 ± 3.0a	22.0 ± 1.2b	3.0 ± 1.8c	0d
*Wickerhamomyces anomalus* YE-42	48.9 ± 0.8a	51.2 ± 1.6a	35.2 ± 1.3b	0c	0c
*Helminthosporium oryzae* DOAC 2293 with					
*Candida michaelii* YE-239	12.6 ± 0.8b	39.6 ± 1.9a	20.9 ± 0.8b	5.7 ± 1.4c	0d
*Hannaella sinensis* YE-19	1.0 ± 1.2d	51.2 ± 1.4a	39.1 ± 1.1b	18.2 ± 0.9c	2.3 ± 2.4d
*Hannaella sinensis* YE-58	0.7 ± 1.3d	49.0 ± 3.5a	23.7 ± 0.7b	7.5 ± 2.1c	0d
*Hannaella hubeiensis* YE-21	11.0 ± 0.2bc	39.2 ± 1.8a	21.1 ± 1.1b	11.9 ± 1.8c	0d
*Rhodotorula mucilaginosa* YE-171	9.3 ± 1.0c	49.7 ± 2.5a	26.8 ± 1.8b	7.0 ± 1.4c	0.5 ± 1.4d
*Wickerhamomyces anomalus* YE-42	25.1 ± 1.0a	40.0 ± 1.2a	22.0 ± 1.8b	17.1 ± 0.7c	0d

^1^ Inhibition (%) = (Diameter of fungal colony grow alone—Diameter of fungal colony grow with yeast/Diameter of colony grow alone) ×100; Each value represents a mean “ ± ” standard deviation (SD). In the same column for each rice pathogenic fungus tested data followed by the different lower-case letters are significantly different according to Duncan’s multiple range test at *p* ≤ 0.05. ^2^ Inhibition (%) = (Radius of control fungal colony—Radius of fungal colony grow with yeast/Radius of control fungal colony) ×100; Each value represents a mean “ ± ” standard deviation (SD). In the same row data followed by the different lower-case letters are significantly different according to Duncan’s multiple range test at *p* ≤ 0.05. ^3^ Standard nutrient concentration (39 g/L PDA powder). ^4^ Half of standard nutrient concentration (19.5 g/L PDA powder). ^5^ One-fourth of standard nutrient concentration (9.7 g/L PDA powder). ^6^ One-tenth of standard nutrient concentration (3.9 g/L PDA powder).

**Table 2:** Activities of cell wall lytic enzymes produced by antagonistic yeasts.

Yeast	Enzyme Activities (mU/mL)	
	Glucanase	Chitinase
*Candida michaelii* YE-239	62.6 ± 0.8	6.0 ± 18.3
*Candida tropicalis* YE-111	102.4 ± 3.0	16.9 ± 29.5
*Hannaella sinensis* DMKU-CP437	0.2 ± 0.0	35.2 ± 3.5
*Hannaella sinensis* YE-19	8.5 ± 3.1	3.8 ± 3.8
*Hannaella sinensis* YE-58	0.2 ± 0.5	0
*Papiliotrema japonica* YE-135	112.9 ± 0.8	504.5 ± 148.4
*Pseudozyma hubiensis* YE-21	27.5 ± 1.8	0
*Rhodotorula mucilaginosa* YE-171	116.5 ± 1.9	173.4 ± 17.7
*Rhodotorula taiwanensis* YE-9	58.9 ± 1.5	1.0 ± 3.2
*Rhodotorula taiwanensis* YE-213	0	0
*Wickerhamomyces anomalus* DMKU-CP122	30.3 ± 4.5	45.2 ± 1.8
*Wickerhamomyces anomalus* DMKU-CP127	0	0
*Wickerhamomyces anomalus* YE-42	48.9 ± 3.5	0

**Table 3:** Biofilm formation, siderophore production, and solubilization of zinc oxide and phosphate by antagonistic yeasts.

Yeast	Biofilm formation			Siderophore Production ^4^	SE ^e^	
	OD_T_^1^	OD Value ^2^	Sum ^3^		Ca_3_(PO)_4_	ZnO
*Candida michaelii* YE-239	0.0973 ± 0.0023	1.68	weak	0	0	0
*Candida tropicalis* YE-111	0.3025 ± 0.0085	5.21	strong	0	0	0
*Hannaella sinensis* DMKU-CP437	0.2296 ± 0.0141	3.96	moderate	0	0	0
*Hannaella sinensis* YE-19	0.2054 ± 0.0096	3.54	moderate	0	0	0
*Hannaella sinensis* YE-58	0.2130 ± 0.0133	3.67	moderate	0	0	0
*Papiliotrema japonica* YE-135	0.1263 ± 0.0026	2.18	moderate	0	0	0
*Pseudozyma hubiensis* YE-21	0.1155 ± 0.0065	1.99	weak	46.5 ± 1.2	0	0
*Rhodotorula mucilaginosa* YE-171	0.3586 ± 0.0194	6.18	weak	9.1 ± 0.2	0	0
*Rhodotorula taiwanensis* YE-9	0.2389 ± 0.0060	4.12	weak	15.5 ± 0.5	0	0
*Rhodotorula taiwanensis* YE-213	0.2062 ± 0.0130	3.56	moderate	14.1 ± 0.1	0	0
*Wickerhamomyces anomalus* DMKU-CP122	0.1831 ± 0.0156	3.16	moderate	32.8 ± 1.1	1.3 ± 0.1	1.3 ± 0.0
*Wickerhamomyces anomalus* DMKU-CP127	0.1331 ± 0.0057	2.29	moderate	32.4 ± 1.6	1.4 ± 0.0	1.3 ± 0.0
*Wickerhamomyces anomalus* YE-42	0.2143 ± 0.0058	3.69	moderate	34.5 ± 0.6	1.5 ± 0.1	1.4 ± 0.1

^1^ The optical density at 620 nm of biofilm layer stained with crystal violet (OD_T_), data express as average OD_T_ ± SD). ^2^ Average optical density of biofilm layer (OD_T_) as a portion of optical density of control (OD_C_). ^3^ Interpretation of biofilm formation: OD_T_ ≤ Ac, no biofilm formation; weak biofilm producer (OD_C_ < OD_T_ ≤ 2 OD_C_), moderate biofilm producer (2 OD_C_ < OD_T_ ≤ 4 OD_C_) and strong biofilm producer (4 OD_C_ < OD_T_), when OD_C_ = 0.0580). ^4^ Diameter of holo zone (cm). ^e^ Solubilization efficiency (SE) = Diameter of the halo zone (cm)/Diameter of the colony (cm).

**Table 4:** Control of rice seedling rot disease caused by *Curvularia lunata* DOAC 2313 by antagonistic yeasts and chemical fungicides.

Treatment	Stem (cm)	Root (cm)	Dry Weight (g) ^2^	Seed Germination (%)	Seedling Vigor Index	Disease Incidence (%)	Disease Control (%)
Negative control	51.87 ± 6.75abc	10.70 ± 4.22cd	120.09 ± 6.63abc	89.00 ± 2.45a	5569.73 ± 218.38a	-	-
Positive control: *Curvularia lunata* DOAC 2313 alone	46.02 ± 7.17i	8.48 ± 3.49e	71.73 ± 7.25d	71.00 ± 4.90gh	3869.67 ± 177.95g	30.52	-
*Curvularia lunata* DOAC 2313 with							
*Candida tropicalis* YE-111	47.86 ± 6.88h	11.01 ± 3.92bcd	86.19 ± 11.34d	77.67 ± 4.19cdefg	4572.33 ± 325.00ef	17.91	41.33
*Hannaella sinensis* YE-19	49.27 ± 7.18efg	12.01 ± 4.52a	110.55 ± 7.88abc	83.00 ± 3.27abcde	5085.67 ± 226.99abcde	8.69	71.52
*Hannaella sinensis* YE-58	49.65 ± 6.87ef	11.31 ± 4.08abcd	105.28 ± 8.00bc	75.33 ± 3.40efg	4592.33 ± 242.28def	17.55	42.50
*Hannaella sinensis* DMKU-CP437	50.91 ± 5.85cd	11.32 ± 4.15abcd	84.68 ± 10.29d	74.67 ± 3.68fg	4646.67 ± 289.27cdef	16.57	45.70
*Kodamaea ohmeri* DMKU-RP06 ^1^	50.37 ± 6.23de	11.54 ± 3.98abc	104.89 ± 6.62bc	81.67 ± 3.40abcdef	5055.67 ± 166.01abcde	9.23	69.76
*Kodamaea ohmeri* DMKU-RP57 ^1^	51.65 ± 6.04abc	11.00 ± 4.03bcd	102.30 ± 5.70c	82.33 ± 3.68abcdef	5158.33 ± 233.27abcde	7.39	75.80
*Kodamaea ohmeri* DMKU-RP233 ^1^	51.44 ± 5.86abcd	11.34 ± 3.83abc	116.72 ± 4.35abc	86.33 ± 2.87ab	5419.33 ± 172.56a	2.70	91.15
*Meyerozyma caribbica* DMKU-RP07 ^1^	52.23 ± 6.34ab	11.15 ± 4.27abcd	114.81 ± 2.93abc	83.00 ± 2.16abcdef	5259.37 ± 147.95abc	5.57	81.74
*Papiliotrema japonica* YE-135	52.10 ± 5.21abc	10.99 ± 3.77bcd	109.54 ± 3.67abc	81.00 ± 1.63abcdef	5110.00 ± 25.66abcde	8.25	72.96
*Rhodotorula mucilaginosa* YE-171	51.20 ± 6.82bcd	10.85 ± 3.68cd	116.82 ± 11.73abc	85.67 ± 3.68abc	5316.00 ± 250.50ab	4.56	85.07
*Rhodotorula taiwanensis* YE-9	48.16 ± 7.25gh	10.44 ± 4.16d	84.76 ± 5.02d	75.67 ± 4.92defg	4434.00 ± 326.70f	20.39	33.19
*Rhodotorula taiwanensis* YE-213	48.58 ± 6.81fgh	11.58 ± 4.02abc	82.95 ± 7.59d	78.67 ± 3.30bcdefg	4733.70 ± 232.82bcdef	15.02	50.78
*Torulaspora indica* DMKU-RP31 ^1^	52.18 ± 4.87abc	11.16 ± 4.05abcd	122.91 ± 8.80ab	88.33 ± 2.49a	5595.00 ± 302.03a	0	100
*Torulaspora indica* DMKU-RP35 ^1^	52.24 ± 5.25ab	10.84 ± 3.75cd	114.63 ± 3.07abc	83.67 ± 2.49abcde	5277.67 ± 179.00ab	5.24	82.82
*Wickerhamomyces anomalus* DMKU-CP122	51.98 ± 5.44abc	11.43 ± 3.93abc	105.23 ± 8.43bc	82.00 ± 4.55abcdef	5199.67 ± 472.67abcd	6.64	78.23
*Wickerhamomyces anomalus* DMKU-CP127	52.52 ± 5.00a	11.43 ± 4.42abc	115.15 ± 3.52abc	83.67 ± 3.30abcde	5350.33 ± 358.39ab	3.94	87.09
*Wickerhamomyces anomalus* DMKU-RP04 ^1^	52.48 ± 4.90ab	11.12 ± 3.91bcd	115.81 ± 2.15abc	85.67 ± 4.99abc	5448.67 ± 349.44a	2.17	92.88
*Wickerhamomyces anomalus* DMKU-RP25 ^1^	51.47 ± 5.98abcd	11.20 ± 3.99abcd	112.51 ± 11.4abc	84.00 ± 2.16abcd	5264.33 ± 172.62abc	5.48	82.03
*Wickerhamomyces anomalus* YE-42	52.45 ± 4.60ab	11.26 ± 3.84abcd	121.52 ± 10.59ab	88.67 ± 2.87a	5649.00 ± 338.01a	0	100
Carbendazim^®^	52.61 ± 5.55a	11.84 ± 4.13ab	127.73 ± 4.54a	81.00 ± 2.94abcdef	5220.67 ± 239.98abc	6.27	79.47
Mancozeb^®^	48.78 ± 6.55fgh	11.10 ± 4.67bcd	79.94 ± 12.34d	64.67 ± 3.68h	3872.33 ± 183.03g	30.48	0.15

Each value represents a mean “ ± ” standard deviation (SD). In the same column, data followed by the different lower-case letters are significantly different according to Duncan’s multiple range test at *p* ≤ 0.05. ^1^ Antagonistic yeasts from previous investigation [13]. ^2^ Dry weight of 100 rice plants nd = not determined.

**Table 5:** Control of rice seedling rot disease caused by *Helminthosporium oryzae* DOAC 2293 by antagonistic yeasts and chemical fungicides.

Treatment	Stem (cm)	Root (cm)	Dry Weight (g) ^2^	Seed Germination (%)	Seedling Vigor Index	Disease Incidence (%)	Disease Control (%)
Negative control	51.87 ± 6.75a	10.70 ± 4.22d	120.09 ± 6.63ab	89.00 ± 2.45a	5544.00 ± 209.93ab	nd	nd
Positive control: *Helminthosporium oryzae* DOAC 2293 alone	45.84 ± 6.95d	8.53 ± 3.56e	82.57 ± 3.31e	76.00 ± 1.63e	4132.00 ± 120.78e	25.47	nd
*Helminthosporium oryzae oryzae* DOAC 2293 with							
*Candida michaelii* YE-239	52.44 ± 5.47a	12.15 ± 8.12a	103.56 ± 1.40cd	81.33 ± 1.70bcde	5253.67 ± 97.04bc	5.24	79.44
*Hannaella sinensis* YE-19	49.07 ± 6.17c	10.52 ± 3.95d	95.27 ± 2.14d	80.00 ± 2.16cde	4766.67 ± 88.21d	14.02	44.95
*Hannaella sinensis* YE-58	52.38 ± 4.46a	11.25 ± 4.07bcd	94.47 ± 1.41d	79.00 ± 2.16de	5027.00 ± 92.61cd	9.33	63.37
*Meyerozyma caribbica* DMKU-RP55 ^1^	52.06 ± 6.15a	11.03 ± 4.30bcd	101.86 ± 3.84d	82.33 ± 2.87bcd	5195.00 ± 194.37bc	6.30	75.27
*Pseudozyma hubiensis* YE-21	50.72 ± 6.05b	10.78 ± 4.03cd	105.60 ± 0.84cd	86.67 ± 2.49ab	5330.33 ± 209.48abc	3.85	84.88
*Rhodotorula mucilaginosa* YE-171	52.35 ± 5.27a	10.92 ± 3.68bcd	94.97 ± 2.12d	78.67 ± 2.87de	4977.33 ± 142.64cd	10.22	59.87
*Torulaspora indica* DMKU-RP31 ^1^	52.24 ± 4.95a	11.08 ± 4.06bcd	123.22 ± 0.58ab	90.33 ± 0.94a	5719.33 ± 120.63a	0	100
*Torulaspora indica* DMKU-RP35 ^1^	52.37 ± 5.17a	10.94 ± 3.83bcd	117.15 ± 3.36ab	87.00 ± 2.94ab	5508.00 ± 247.92ab	0.65	97.45
*Wickerhamomyces anomalus* DMKU-RP04 ^1^	52.26 ± 4.94a	10.77 ± 3.92cd	118.11 ± 3.99ab	88.33 ± 2.05a	5567.33 ± 200.73ab	0	100
*Wickerhamomyces anomalus* DMKU-RP25 ^1^	51.75 ± 5.62a	11.27 ± 4.14bcd	109.58 ± 3.27bc	85.00 ± 1.63abc	5357.33 ± 121.26abc	3.37	86.77
*Wickerhamomyces anomalus* YE-42	52.39 ± 4.82a	11.65 ± 4.24abc	121.85 ± 7.20ab	89.00 ± 1.41a	5699.33 ± 189.84a	0	100
Carbendazim^®^	52.59 ± 5.57a	11.85 ± 4.06ab	127.73 ± 4.54a	84.67 ± 3.68abc	5445.67 ± 238.94ab	1.77	94.04
Mancozeb^®^	48.83 ± 6.51c	11.22 ± 4.61bcd	79.94 ± 8.34e	69.67 ± 4.11f	4175.92 ± 265.19e	24.69	3.05

Each value represents a mean “ ± ” standard deviation (SD). In the same column, data followed by the different lower-case letters are significantly different according to Duncan’s multiple range test at *p* ≤ 0.05. ^1^ Antagonistic yeasts from previous investigation [13]. ^2^ Dry weight of 100 rice plants. nd = not determined.

## Supplementary Materials

The following are available online at https://www.mdpi.com/2076-2607/8/5/647/s1.
