# Supplementary material for: Biocontrol of Rice Seedling Rot Disease Caused by Curvularia lunata and Helminthosporium oryzae by Epiphytic Yeasts from Plant Leaves
Source: Microorganisms. 2020 Apr 29;8(5):647. doi: 10.3390/microorganisms8050647 (PMC7285343; doi:10.3390/microorganisms8050647)
Supplement: Supplementary file 1 [file microorganisms-08-00647-s001.pdf]

Table S1. Yeast species and strains belonging to the phylum Ascomycota and Basidiomycota used in this study

Taxa	Strain	No. of strain
**Phylum Ascomycota, Subphylum Saccharomycotina**		
Blastobotrys sp.	YE-8	1
Candida fermentati	DMKU-CP175, DMKU-CP265, DMKU-CP373, DMKU-CP79, DMKU-CP367	5
Candida floricola	DMKU-CP149, DMKU-CP367	2
Candida fukuyamaensis	DMKU-CP312	1
Candida intermidia	DMKU-CP07, DMKU-CP03, DMKU-CP377, DMKU-CP57, DMKU-CP59, YE-20	6
Candida michaelii	YE-239	1
Candida nivariensis	DMKU-CP497	1
Candida parapsilosis	DMKU-RP300	1
Candida pseudointermedia	DMKU-CP501	1
Candida tropicalis	YE-111, YE-115	2
Candida wangnamhkiaoensi	YE-166	1
Hanseniaspora guilliermondii	DMKU-CP295	1
Hanseniaspora opuntiae	DMKU-CP99	1
Hyphopichia burtonii	YE-164	1
Kodamaea ohmeri	DMKU-CP258, DMKU-CP281, DMKU-CP471, DMKU-CP51	4
Meyerozyma caribbica	DMKU-CP140, DMKU-CP160, DMKU-CP43, DMKU-CP48, DMKU-CP503, DMKU-CP202, DMKU-CP216, YE-184, YE-201, YE-238, YE-240	11
Meyerozyma guilliermondii	YE-100	1
Pichia myanmerensis	YE-165	1
Wickerhamomyces anomalus	DMKU-CP122, DMKU-CP127, YE-42	4
**Phylum Basidiomycota, Subphylum Agaricomycotina**		
Goffeauzyma iberica	DMKU-CP72	1
Kwoniella dendrophila	DMKU-CP263	1
Kwoniella haveanensis	DMKU-CP60, DMKU-CP96, DMKU-CP222	3
Hannaell coprosmaensis	DMKU-CP350	1
Hannella luteola	DMKU-CP282, DMKU-CP469	2
Hannella oryzae	DMKU-CP177, DMKU-CP174, YE-69	3
Hannella sinensis	DMKU-CP181, DMKU-CP184, DMKU-CP188, DMKU-CP294, DMKU-CP299, DMKU-CP335, DMKU-CP321, DMKU-CP386, DMKU-CP430, DMKU-CP437, YE-19, YE-56, YE-58	13
Hannella siamensis	DMKU-CP200, DMKU-CP09, YE-124	3
Papiliotrema aspenensis	YE-105	1
Papiliotrema flavescens	YE-127	1
Papiliotrema hoabinhensis	DMKU-CP97	1
Papiliotrema japonica	YE-135	1
Papiliotrema laurentii	DMKU-CP250, YE-23	2
Papiliotrema nemorosus	DMKU-CP380, DMKU-CP391	2
Papiliotrema rajasthanensis	DMKU-CP395, DMKU-CP02, DMKU-CP10, DMKU-CP204, DMKU-CP213, DMKU-CP348 DMKU-CP502, DMKU-RP222, YE-253	9
Papiliotrema siamense	YE-43	1

Taxa	Strain	No. of strain
Saitozyma flava	DMKU-CP150	1
Trichosporon asahii	YE-116	1
Trichosporon asteroides	YE-169	1
**Phylum Basidiomycota, Subphylum Pucciniomycotina**		
Occultifer externus	DMKU-CP332	1
Rhodosporiobolus fluvialis	DMKU-CP34, DMKU-CP293	2
Rhodosporiobolus ruineniae	DMKU-CP270, DMKU-CP284, DMKU-CP353, DMKU-CP355	4
Rhodotorula mucilaginosa	DMKU-CP165, YE-102, YE-112, YE-171, YE-175	5
Rhodotorula paludigena	DMKU-CP168, DMKU-CP234, DMKU-CP256, DMKU-CP259, DMKU-CP493, DMKU-CP119, DMKU-CP162, DMKU-CP170, DMKU-CP179, DMKU-CP203, DMKU-CP218, DMKU-CP411, DMKU-CP460, YE-52	14
Rhodotorula slooffiae	DMKU-CP485, DMKU-CP135,	2
Rhodotorula sp.	DMKU-CP209, DMKU-CP246, DMKU-CP297, YE-188	4
Rhodotorula taiwanensis	DMKU-CP04, DMKU-CP18, DMKU-CP55, YE-126, YE-213, YE-9	6
Rhodotorula toruloides	DMKU-CP474	1
Sporidiobolus pararoseus	DMKU-CP195, DMKU-CP183, DMKU-CP186, DMKU-CP305, DMKU-CP322, DMKU-CP403, DMKU-CP418, DMKU-CP505, DMKU-CP513, YE-17, YE-249, YE-60	12
Sporobolomyces bannaensis	DMKU-CP24	1
Sporobolomyces blumeae	YE-144, YE-15	2
Sporobolomyces nylandii	DMKU-CP133, DMKU-CP217, DMKU-CP230	3
Sporobolomyces carnicolor	DMKU-CP29, DMKU-CP477, DMKU-CP489	3
Sporobolomyces sp.	YE-81	1
Symmetrospora marina	DMKU-CP155, DMKU-CP149	2
Symmetrospora oryzicola	DMKU-CP457	1
Symmetrospora suhii	DMKU-CP145, DMKU-CP443	2
**Phylum Basidiomycota, Subphylum Ustilaginomycotina**		
Dirkmeia churashimeansis	YE-223, YE-71	2
Moesziomyces aphidis	DMKU-CP111, DMKU-CP220	2
Moesziomyces antarcticus	DMKU-CP124, YE-142	2
Pseudozyma hubeiensis	YE-21	1
